# Role of Different Enzymes in H_2_O_2_ Neutralization and Cellular Radioresistance, Estimated by Mathematical Modeling

**DOI:** 10.3390/ijms26167754

**Published:** 2025-08-11

**Authors:** Sylwia Ciesielska, Krzysztof Mazur, Krzysztof Fujarewicz, Joanna Rzeszowska-Wolny

**Affiliations:** 1 Department of Systems Biology and Engineering, Faculty of Automatic Control, Electronics and Computer Science, Silesian University of Technology, 44-100 Gliwice, Poland; krzysztof.fujarewicz@polsl.pl (K.F.); joanna.rzeszowska@polsl.pl (J.R.-W.); 2 Biotechnology Centre, Silesian University of Technology, 44-100 Gliwice, Poland; 3 Department of Measurements and Control Systems, Faculty of Automatic Control, Electronics and Computer Science, Silesian University of Technology, 44-100 Gliwice, Poland; krzysztof.jan.mazur@polsl.pl

**Keywords:** reactive oxygen species, H_2_O_2_ neutralization, ROS neutralization mathematical modelling

## Abstract

Reactive oxygen species (ROS) are fundamental components found in cells that exist in an oxygen environment. While they are often viewed as detrimental metabolic byproducts that can harm cells, leading to aging and cell death, they can also play a role in cellular regulatory processes and have beneficial effects. One of the main ROS present in all cells is hydrogen peroxide (H_2_O_2_), which can function as a signaling molecule in extra- and intracellular signaling. To enhance our understanding of how various enzymes regulate cellular H_2_O_2_ levels, we created a mathematical model of H_2_O_2_ neutralization and performed computer simulations to estimate the neutralization efficiency in various types of cells. Data on gene expression for genes participating in this process were incorporated into the calculations, along with the regulation of enzymes in oxidation and reduction processes. The conducted simulations demonstrate that cells originating from different tissues utilize systems neutralizing H_2_O_2_ variously, which results in differences in H_2_O_2_ cellular levels. The simulation findings suggest that the differences in radiosensitivity seen in various cancer cell types may be linked to their effectiveness in scavenging H_2_O_2_. Analysis of results from model simulations for colorectal, lung, and breast cancer cell lines indicated that radiosensitive cell lines exhibited elevated levels of H_2_O_2_, attributed to the reduced efficiency of neutralizing enzymes. By highlighting cell-type-specific differences in H_2_O_2_ neutralization, our findings may contribute to a deeper understanding of redox regulation in cancer cells and reveal new potential correlations with radioresistance.

## 1. Introduction

Reactive oxygen species (ROS) are generally regarded as harmful byproducts resulting from life in an oxygen environment [1]. They are recognized as secondary messengers in overall cellular signaling [1,2]. For a long time, these species were thought to be damaging to cells and involved in aging and cell death [1,3,4]. Today, they are seen as crucial players in the regulation of cellular processes such as proliferation, senescence, and apoptosis [3,5]. Hydrogen peroxide (H_2_O_2_) is a non-radical member of the reactive oxygen species group, which occurs in large amounts in cells of different organisms, including plants [6,7]. H_2_O_2_ concentration in cells has been shown to vary and is assumed to be 1–700 nM [8,9], with cytotoxic values inducing apoptosis over 700 nM [8,9]. It should be noted that the actual intracellular concentration of H_2_O_2_ reached in the steady state incubation is lower than the extracellular value that leads to cell death [8]. Thus, the levels of H_2_O_2_ found outside the cell in vitro, which are generally cytotoxic, are significantly elevated compared to the intracellular concentrations of H_2_O_2_ that are assumed to be harmful [4]. Concentrations up to 15 µM have been found to stimulate cell proliferation, whereas those over 1 mM were shown to lead to necrotic cell death. H_2_O_2_ is found to be stable in comparison to other members of the ROS group, with the half-life within milliseconds [6]. Due to its long half-life, it is considered a signaling molecule [10] that is able to influence cellular processes like apoptosis or cell proliferation [6,7]. Hydrogen peroxide is formed by the dismutation of superoxide (O_2_^.−^); it is also generated by enzymes such as amino acid oxidase, xanthine oxidase, and NOX [11,12]. H_2_O_2_ can freely pass through cell membranes and directly induce the breaking of phosphodiester bonds [1,13]. H_2_O_2_ can react with metals, producing OH (Fenton reaction) [14]. It is believed that the greatest toxicity of H_2_O_2_ and O_2_^.−^ is achieved during the transition to hydroxyl radicals [14,15,16].

**Figure 1:**
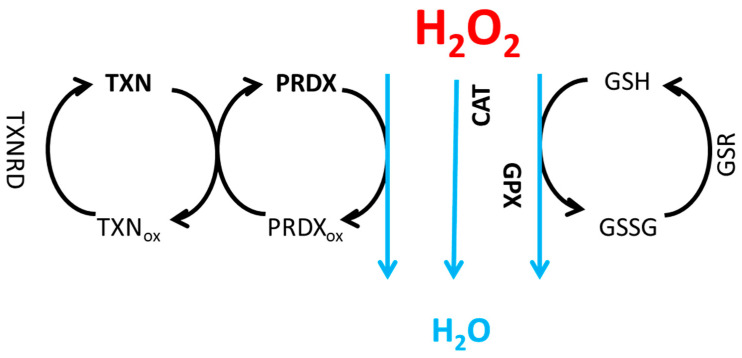
H_2_O_2_ neutralization to water by enzymes: peroxiredoxin (PRDX) with thioredoxin (TXN), catalase (CAT) and glutathione peroxidase (GPX) with glutathione (GSH). PRDX in reduced form is oxidized (PRDXox), TXN is used for re-reduction, which is then oxidized (TXNox) and then reduced again by thioredoxin reductase (TXNRD). In the case of GPX, GSH is used for reduction, which is oxidized to GSSG and then reduced by glutathione reductase (GSR).

Neutralization of hydrogen peroxide and thus protection against damage is carried out mainly by catalase (CAT) [1,13,17], an antioxidant present in almost all aerobic organisms, through the interaction with glutathione peroxidase (GPX), in which glutathione is converted into its oxidized form GSSG [18,19,20], and by enzymes from the peroxiredoxin family (PRDX), which were used for oxidation by the H_2_O_2_ (Figure 1) [21,22,23].

Neutralizing enzymes targeting H_2_O_2_ are evolutionarily conserved from sponges to mammals, with the glutathione-linked GPX system being the youngest neutralizing family. CAT and PRDX are old families which are well conserved between species, with their essential antioxidant functions having led to the conservation of genes throughout the animal kingdom [24]. Catalase exists in three main types: typical catalases (in aerobic organisms), catalase-peroxidases (in fungi, archaea, and bacteria), and manganese catalases (bacteria-specific). Typical catalases are categorized into three subgroups, the third of which encompasses animals, including humans [25,26]. Catalase is commonly found in cells, particularly in peroxisomes (liver cells) and in the cytoplasm (erythrocytes). Besides neutralizing hydrogen peroxide, it also decomposes peroxynitrite and oxidizes nitric oxide to nitrite [20,21]. Glutathione peroxidase functions by using glutathione (GSH) to neutralize ROS. This process relies on the redox cycling of GSH, which is regulated by glutathione reductases and peroxidases. GPX1 is found in the cytosol, nucleus, and mitochondria; GPX2 is located in the cytosol and nucleus; GPX3 is present in the cytosol; GPX4 is distributed in the nucleus, cytosol, mitochondria, and cell membranes [18,27].

Cells require balanced redox conditions to properly function, maintained by strictly controlled oxidation and reduction processes. For the antioxidant system to function correctly, it is necessary to convert oxidized enzymes back to their reduced state. This function is carried out by glutathione reductase (GSR), which catalyzes the reduction of GSSG to GSH [18], and thioredoxins (TXN), which reduce peroxiredoxins [28]. Peroxiredoxins, thioredoxins, and glutathione contain cysteines with thiol groups, which occur in reduced (S-H) or oxidized (S-S) forms. During the reduction of H_2_O_2_, peroxiredoxins are oxidized and later reduced by thioredoxins, which are, in turn, oxidized. To ensure the system works correctly, oxidized thioredoxins must be reduced by thioredoxin reductases (TXNRD), the basic elements of the thioredoxin system, which restore their enzymatic activity [29]. The electrons essential for the reduction of enzymes originate from the constant reduction reactions that occur during metabolism [23,29].

Antioxidant action of H_2_O_2_-neutralizing enzymes varies among organelles. Melo et al. showed that neutralization systems do not function equally in different cellular locations, e.g., in erythrocytes, CAT is more vital for cytoplasmic antioxidant protection than for that of membrane components; when its activity is impaired, PRDX and GPX are transported to the cell membrane, probably to protect against lipid peroxidation [30]. Catalytic activities of the main H_2_O_2_ antioxidant enzymes also differ. PRDX catalytic activity was proven to compete with GPX in H_2_O_2_ neutralization, and the PRDX reaction rate constant was in the range of 10^7^ M^−1^ s^−1^, comparable to catalase, whose reaction rate constant is in the range of 10^7^ M^−1^ s^−1^, whereas for GPX1, it was shown to be ~10^8^ M^−1^ s^−1^ [31].

For a better understanding of the processes observed in cells, mathematical models are used. They, in a simplified way, represent the reactions occurring in cells. Computer simulations make it possible to track many different variants of cell behavior. Regulatory processes of ROS ensure the proper functioning of cells, and a disturbed balance, called oxidative stress, can lead to cell death or its pathology. Differences in the expression of neutralizing enzymes observed in our previous studies and analyses [32,33] suggested that it would be important to be able to predict differences in ROS levels for different cell types of different tissue origins. Since an ROS balance is critical for cellular health and influences processes like cancer progression and treatment responses, predicting how distinct neutralization pathways affect H_2_O_2_ levels is essential. A systematic analysis through a mathematical model to explore how differences in enzyme systems affect H_2_O_2_ dynamics in various cell types could enhance the understanding of the mechanisms behind oxidative stress. There are multiple models in the literature describing systems related to reactive oxygen and nitrogen species [34,35,36,37]. The existing mathematical models related to the neutralization of ROS have focused mainly on the oxidative processes taking place in the mitochondria (see Kembro et al. [28]). Therefore, we created a model describing the process of H_2_O_2_ neutralization regarding the oxidation/reduction of antioxidant enzymes, focused on the differences in neutralization between different types of cells. We conducted a series of computer simulations using publicly available expression data on neutralizing enzymes to compare differences in various cell types with special attention to colorectal, lung, and breast cancers. Using the created model, we also tried to check whether there were differences in H_2_O_2_ neutralization between cell lines of the same origin that differed in the expression of neutralizing enzymes. We considered the influence of radiation and explored relationships between ROS neutralization and radiosensitivity and radioresistance. In the conducted simulations, we focused on neutralization systems with enzymes such as catalase, peroxiredoxin with thioredoxin, and glutathione peroxidase with glutathione, and we showed that these neutralization systems can differentiate radiosensitive and radioresistant cells among colorectal, lung, and breast cancer cell lines.

## 2. Results and Discussion

One of the major reactive oxygen species present in all cells is H_2_O_2_, which can act as a signaling molecule in intracellular and extracellular signaling. Using the created model, we tried to check whether there were differences in H_2_O_2_ neutralization between cell lines in which the expression of neutralizing enzymes was different. Considering the connection between ROS and radiation, we also explored how H_2_O_2_ levels might relate to radiosensitivity and radioresistance. Our objective was to assess whether all three neutralization pathways (PRDX/TXN, CAT, and GPX/GSH) were equally effective in every cell type or if certain preferences existed. In computer simulations, we used a constant supply of H_2_O_2_ to the system, which, in a real system, can be considered as a constant supply from mitochondria, and then we checked how H_2_O_2_ neutralization proceeded by switching off different neutralization systems one by one (such a situation is possible, e.g., in the case of mutation of the enzymes) and for all systems operating correctly. We observed how this mutation influenced the level of H_2_O_2_ in different types of cells.

**Figure 2:**
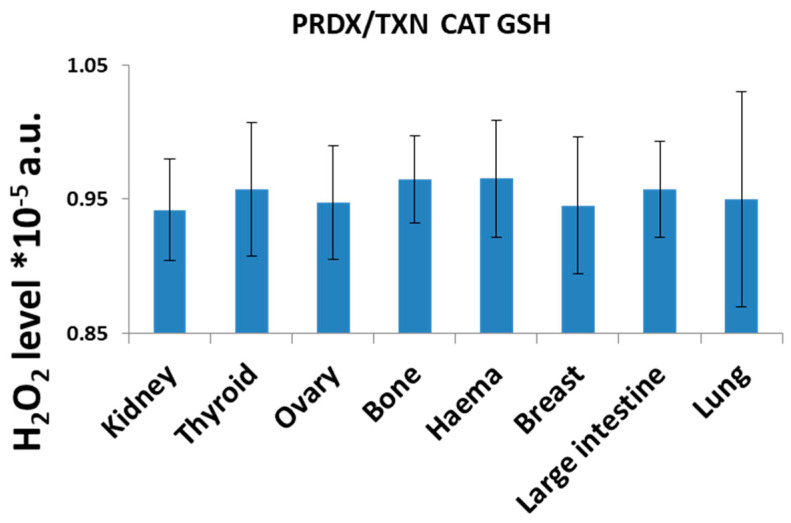
Computer simulations of H_2_O_2_ levels in cell lines of different tissue origin obtained with assumptions that all systems of neutralization are active. The results present average values obtained for different cell lines of the same origin ± SD.

We investigated kidney, thyroid, ovary, bone, hematopoietic, breast, large intestine, and lung cancer cell lines. The results of the H_2_O_2_ neutralization mathematical model are demonstrated in Figure 2, which shows that when all systems were working, there were slight differences in H_2_O_2_ level, and we were not able to distinguish any particular pattern, with the process of neutralization remaining similar in different tissues. When all systems were working, the neutralization of H_2_O_2_ was the least efficient in hematopoietic and bone tissue and the most efficient in breast and kidney tissue.

**Figure 3:**
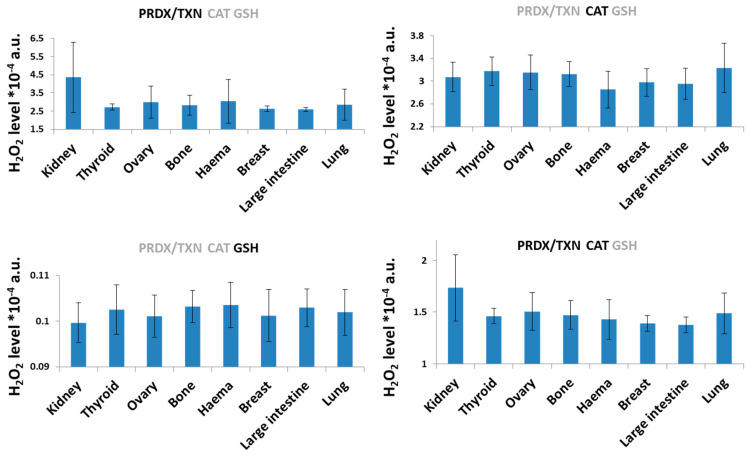
Computer simulations of H_2_O_2_ levels in cell lines of different tissue origin obtained with assumptions that only some of the systems of neutralization are active. Active system of neutralization was marked as black, the grey is the one switched off. The results present average values obtained for different cell lines of the same origin ± SD.

Because there were no particular differences in neutralization processes when all neutralization pathways were switched on, we wanted to check if there were any differences when some of the pathway/s were switched off. Turning off one of the neutralization systems indicated differences among cell lines originating from various tissues, but these differences were only significant for the PRDX/TXN and CAT pathways. The combination of these two systems effectively neutralized H_2_O_2_, particularly in breast and large intestine tissues, while exhibiting the least efficiency in kidney cells (Figure 3). The results for CAT + GSH and PRDX/TXN + GSH were comparable to those of GSH alone, demonstrating that this system was the most effective neutralization pathway in our model, and this is why only this one is included in Figure 3. Such outcomes are observed because GPX/GSH is the most efficient system of neutralization [38] as a result of the significant concentration of GSH that we have assumed in our model. GSH is also extensively used in other cellular processes, suggesting that the actual GSH available for GPX/GSH neutralization is probably much lower.

Analysis of cell lines from different tissue types reveals that the system with only the PRDX/TXN system working was the least efficient in kidney tissue and the most efficient in large intestine and breast tissue. The neutralization pathway of catalase was the least efficient in lung tissue and the most efficient in hematopoietic tissue. The glutathione system was the least efficient in hematopoietic tissue and the most efficient in kidney tissue. Differences among tissues in neutralizing the H_2_O_2_ were observed for different pathways of neutralization.

### H_2_O_2_ Neutralization Pathways and Their Connection to Radioresistance

**Figure 4:**
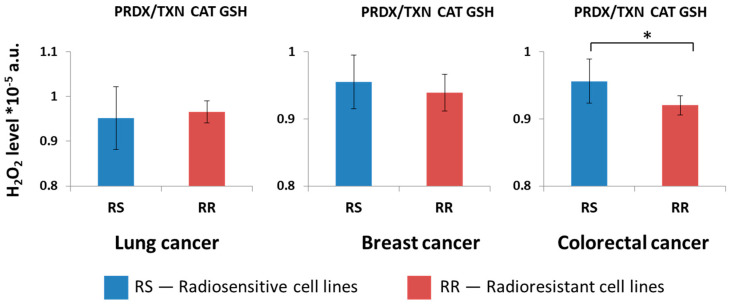
Average H_2_O_2_ level for radiosensitive (RS) and radioresistant (RR) lung, breast and colorectal cancer cell lines with all neutralization systems active obtained in computer simulations. The results are presented as the mean ± SD. Asterisk denote statistical significance with *p*-value < 0.05 comparing radioresistant and radiosensitive cell line groups.

Analyses show the differences among tissues according to the neutralization pathway. Consequently, we aimed to investigate whether there were variations among cell lines derived from the same tissue origin. The analyses were performed on various cell lines from a single tissue type, based on the characterizations documented in the literature. We conducted a series of analyses and selected lung, breast, and colorectal cancer cell lines based on their radiosensitivity. Indeed, there was a difference in radiosensitivity depending on neutralization systems observed in cell lines of these tissues. In colorectal cancer, there was a difference between radiosensitive and radioresistant cell lines; cells could be distinguished due to the GPX-GSH neutralization system, as radioresistant cell lines were more efficient in neutralizing H_2_O_2_. We also observed major differences among kidney cell lines (but this type of cancer is not well characterized in the literature. Some of the analyzed cell lines with less effective peroxiredoxin systems are radioresistant, but due to low data availability, the analyses could not be performed. Figure 4 shows the results with all three systems of neutralization working.

Lung breast, and colorectal cancers are the most frequently diagnosed cancers. Here, we show the simulation results of H_2_O_2_ neutralization for cell lines of these tissue origins. They varied due to their potential for neutralization of H_2_O_2_ by H_2_O_2_-neutralizing enzymes and demonstrated differences between radioresistant and radiosensitive cell lines, which are presented in Table 1.

**Figure 5:**
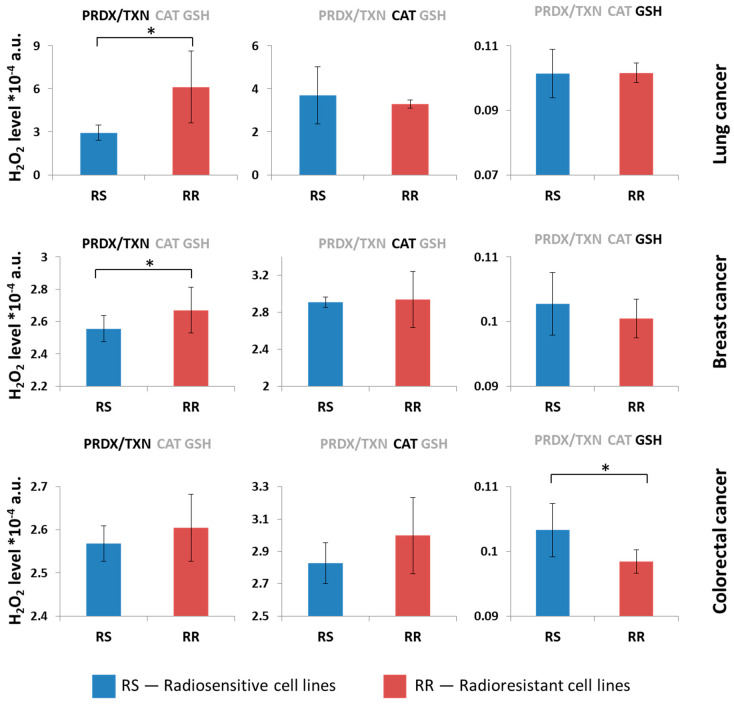
Average H_2_O_2_ level for radiosensitive (RS) and radioresistant (RR) lung cancer cell lines with some of neutralization systems active obtained by computer simulations. First row shows neutralization of H_2_O_2_ in lung cancer, second row in breast cancer and the third in colorectal cancer. Active system of neutralization was marked as black, the grey is the one switched off. The results are presented as the mean ± SD. Asterisk denote statistical significance with *p*-value < 0.05 comparing radioresistant and radiosensitive cell line groups.

Fourteen lung cell lines were classified as radiosensitive (RS) and radioresistant (RR), according to the literature [39,40,41,42,43,44,45,46,47,48]. Sixteen breast cancer cell lines were classified as RS and RR, according to the literature [49,50,51,52,53,54,55,56,57,58,59,60,61]. Twelve radioresistant colorectal cancer cell lines were classified as RS and RR, according to the literature [62,63,64,65,66]. Contrary to other radioresistant colorectal cancer cell lines, SW48 [65,66] seemed to be radiosensitive, with efficient neutralization of H_2_O_2_ by the GPX/GSH system. The results of H_2_O_2_ neutralization in these cells are shown in Figure 5.

Computer simulation results suggest the important role of PRDX/TXN and GPX/GSH systems in the functioning of the cells. In our simulations, cell lines with the less-effective H_2_O_2_ neutralization through the PRDX/TXN system (the highest H_2_O_2_ amount in steady state after neutralization) correlated with the radioresistance of these cells. Significant differences were observed for lung and breast cancer cell lines; in colorectal cancer cell lines, the PRDX/TXN system was also less efficient in radioresistant cells, but this observation was not statistically significant.

Peroxiredoxins (thioredoxin peroxidases) are major thiol-targeting enzymes that represent up to 1% of the protein content in some organisms. Their primary role is to serve as antioxidants for H_2_O_2_ and ONOO-, effectively reducing cellular peroxides by nearly 90% [21]. Six peroxiredoxins can be distinguished: PRDX1, PRDX2, and PRDX6, located in the cytosol, PRDX3, located in mitochondria, PRDX4, located in the extracellular space, and PRDX5, located in mitochondria and peroxisomes [23]. Results from Ref. [65] indicate that higher PRDX1 and PRDX2 mRNA levels are linked to better survival in lung cancer, while PRDX5 and PRDX6 are associated with worse outcomes [68]. H_2_O_2_ added to cells can block antioxidant enzymes through microRNA action for a specific window of H_2_O_2_ doses [69]. Such a response to oxidant exposure is rather counterintuitive. However, H_2_O_2_ acts as a signaling molecule, and the observed changes may be elements of the establishment of H_2_O_2_ levels that are specific and optimal for a given cell type. Indeed, H_2_O_2_ acts as a promoter for cell-cycle progression by oxidizing specific thiol proteins [70]. This group includes PRDXs that serve as signaling mediators, enabling the local accumulation of H_2_O_2_ through the inactivation of their peroxidase function [70]. Rising H_2_O_2_ levels can overoxidize PRDXs, limiting their scavenging ability [70]. The concentrations of intracellular H_2_O_2_ increase as the cell cycle progresses from G1 to mitosis [70]. Cells are the most radiosensitive in G2/M phase of the cell cycle; however, G2/M cell-cycle arrest was shown to correlate with the radioresistance of cells [71,72]. Our findings of increased H_2_O_2_ after PRDX/TXN neutralization need further studying, as PRDXs may prevent premature cell-cycle progression under oxidative stress from UV or IR during the interphase [70], the knockdown of PRDX2-sensitized glioma cells to IR and its decrease, lowered GSH and GSR activity, increased cell-cycle doubling time, and reduced clonogenic cell survival after IR and H_2_O_2_ [73]. 

Neutralization by catalase is comparable in lung and breast cancer cells and less effective in radioresistant colorectal cancer cell lines; however, it is not statistically significant. D-penicillamine (DPEN) with copper can generate H_2_O_2_ in cancer cells and induce clonogenic cell-killing, while catalase inhibited this effect [74]. H_2_O_2_ induced apoptosis in leukemia KG1 cells, likely via catalase deactivation, ROS accumulation, ATP depletion, caspase-3 activation, and altered Bcl-2 family expression [75]. According to the findings in Ref. [76], different concentrations of H_2_O_2_ can affect both cell growth and programmed cell death. At 50–200 μM, the growth of MCF-7 breast cancer cells was inhibited; 1–10 μM stimulated hepatoma 7721 cell growth, and 10 μM increased HT-29 colon cancer cell proliferation. A high dose (1000 μM) induced apoptosis, while 100 μM H_2_O_2_ reduced the migration of H460 lung cancer cells [76].

We also observed the difference for the GPX/GSH system of neutralization (a similar pattern to all enzymes active; see Figure 4). In colorectal cancer, radioresistant cells are significantly more effective in H_2_O_2_ neutralization than radiosensitive cells, and similarly, on average, in breast cancer cells; however, this difference is not statistically significant. In lung cancer, neutralization by the GPX/GSH pathway is comparable. In the research of Zhang et al., they showed that radioresistant cell lines have lower levels of ROS and sensitization of LS180 cells, with GNP-PEG and GNP-PEG-R8 increased ROS and induced apoptosis [77]. In these studies, ROS was marked with dye DCFH-DA, which detects several radicals; however, it was first used for the detection of H_2_O_2_ [77,78,79] so observed changes may reflect mainly H_2_O_2_ levels that correspond with our findings. Similar to our simulation results, lower ROS levels in colorectal cancer were connected to radioresistance, and higher levels were connected to radiosensitivity [77]. In Ref. [80], researchers demonstrated that radioresistant cell lines, developed through repeated irradiation, had lower ROS levels and increased expression of cell viability genes compared to wild-type cells [80]. GSH significantly influenced cell-cycle progression, with its levels changing during the cycle; the highest concentrations of GSH were detected in the G2 and M phases, intermediate levels were noted in the S phase, and the lowest levels were seen in the G1 phase of the cell cycle [81]. Analogous to colorectal cancer, cancer stem cells (CSCs) overexpressed genes involved in GSH synthesis. Depletion of these genes resulted in the elevation of ROS levels and reduced colony formation in CSCs [82]. Depletion of GSH itself enhanced the radiosensitivity of CSCs. Emmink et al. found that GPX2 downregulation increased ROS, increased sensitivity to H_2_O_2_-induced apoptosis, and reduced colon cancer cell growth and metastasis, suggesting ROS elevation could improve chemotherapy responses [83]. The same pattern regarding ROS was observed in head and neck cancers; MitoTam treatment raised ROS levels and increased cell death, even in radioresistant UT-SCC-5 cells [84]. Park et al. showed that H_2_O_2_ inhibited lung cancer cell growth by inducing cell death and G1-phase arrest in Calu-6 and A549 cells, but not in HeLa cells; the effect varied by cell type and H_2_O_2_ concentration [85]. It appears that H_2_O_2_ and its neutralization enzyme levels fluctuate regularly and are directly related to the cell cycle, which may impact the radioresistance of particular cells.

Cancer cells produce higher levels of reactive oxygen species (ROS) due to an increased metabolic rate, genetic mutations, and hypoxia. Studies demonstrated that cancer cells can adapt to elevated ROS levels by activating antioxidant pathways, which enhance their ability to neutralize ROS [86]. A reduced level of ROS in tumor cells correlates with enhanced radioresistance [82]. However, H_2_O_2_ is used in therapy to sensitize radioresistant cells. Good sensitization effects were observed for melanoma [87] and cervical, liver, and breast cancer [88]. Panieri et al. showed that resistant NSCLC cells can be sensitized by high H_2_O_2_ levels (48 μM), causing DNA damage and ATP depletion via a caspase-independent pathway, or by lower levels (6.5 μM), which inhibit glycolysis and ATP recovery [77]. Antioxidant enzyme expression differs across various cell types, and the neutralization process does not contribute equally in all cell types. As a result, different levels of reactive oxygen species (ROS) are observed in cells, which may relate to the differing effects of H_2_O_2_ at various concentrations, particularly on apoptosis and cell proliferation. It seems that cell regulation depends on the intensity of oxidative stress. At lower H_2_O_2_ concentrations, cells appear to increase the production of stress-protective proteins, including antioxidants, to actively neutralize ROS. In contrast, higher H_2_O_2_ levels may promote a shift toward recovery strategies, such as enhanced ribosome association with key mRNAs, enabling rapid protein synthesis once oxidative stress is removed [89].

While our model offers a qualitative perspective and is based on a simplified description of the H_2_O_2_ scavenging process, as outlined in the Materials and Methods section, it still enables valuable predictions and highlights certain trends. Data from our model analyzing cell lines of various tissue origins demonstrate that neutralization systems can differentiate cells both within the same tissue origin and across different origins. This capability is especially relevant due to the significant role of ROS generated during anticancer therapies involving ionizing radiation, which induces ROS, leading to changes in cells. In irradiated cells, there are observed changes in gene expression, the inhibition of proliferation, and the death rate. However, some cells do not respond to treatment, and those that avoid death are called radioresistant [90]. The effectiveness of radiation therapy in curing human tumors varies greatly. This variability in treatment has been linked to several factors, including the presence of hypoxic cells, inadequate reoxygenation during therapy, low intrinsic radiosensitivity, and the potential for repairing lethal damage [91]. Cells that are mature, differentiated, and not actively dividing (e.g., neurons) are more radioresistant. A cell that is radiosensitive would be more prone to die after exposure to ionizing radiation than a radioresistant one [92].

Findings from the simulations imply that the disparities in radiosensitivity across various cancer cell types could be related to their capacity to neutralize H_2_O_2_. Analysis of colorectal, lung, and breast cancer cell lines showed that radiosensitive cells have different levels of H_2_O_2_ than radioresistant cell lines, which may be due to the decreased effectiveness of neutralizing enzymes. Previous analyses [32,33], together with data collected from the model, confirm the importance of H_2_O_2_, suggesting that it might be a key molecule for understanding radioresistance in cancer cells. Neutralization capacity might serve as a biomarker or target for radiosensitization. However, the exact role of H_2_O_2_ in this process remains to be elucidated.

## 3. Materials and Methods

In our previous research, we found that different cell types exhibited distinct levels of antioxidant enzymes. We concluded that the neutralization process can occur via different leading pathways in those cells, which explains the observed differences in various kinds of ROS levels among these cells. The aim of the introduced model was to analyze how the levels of H_2_O_2_ were affected by different neutralization systems. We simulated the neutralization process and checked whether there were differences between the H_2_O_2_ neutralization systems using the following enzymes: peroxiredoxin (PRDX) with thioredoxin (TXN), glutathione peroxidase (GPX) using glutathione, and catalase (CAT). The first two systems were complex and required several oxidation and reduction reactions, which is why we included these dependencies in the model. To better understand the neutralization process, we analyzed multiple cell lines from different tissue origins available in the Cancer Cell Line Encyclopedia (CCLE) database based on RNA-seq data [93]. The analysis was performed for 1025 cancer cell lines from various tissues, and we simulated their neutralization of H_2_O_2_. All necessary information about cell lines used in CCLE database are included [93]. Figure 1 presents the process of H_2_O_2_ neutralization through different pathways: the first pathway was PRDX/TXN, the second was CAT, and the third was the GPX-GSH pathway.

We aimed to determine whether the three neutralization pathways (PRDX/TXN, CAT, and GPX/GSH) were engaged in the neutralization process in all cell types with comparable effectiveness, or if there were specific preferences among different cell lines. We examined this by disabling certain pathways, and one of them remained operational. It is crucial to understand that in living cells, the levels of H_2_O_2_ and other ROS fluctuate, and our model simplifies this by representing an average ROS level at a given moment. The dynamic fluctuations of ROS, which are closely related to their behavior, are not accounted for. Instead, we implemented oxidation and reduction processes, which are crucial components in maintaining a redox balance in cells. The equations for the mathematical model were implemented in the Matlab Simulink environment, where all simulations of the model equations were performed. The version of MATLAB used was R2021a. The created model was used to conduct a series of computer simulations.

Values of enzyme concentrations in multiple cell lines were hard to obtain due to practical issues; therefore, the data for the model were sourced from existing literature and represent values obtained from various cell lines. These data were adapted from Refs. [34,35,94,95,96,97] and presented in Table 2. Model parameters were obtained from Refs. [34,35] through the linearization of equations presented in those papers in operating points to quantify proper values for equation coefficients used in the model presented in Table 3. Rate constants (k) show the speed of processes and how quickly the process happens. Our model assumed that the reaction parameters remained the same for different cell types, but the expression of neutralizing enzymes varied between cells. The levels of enzymes were estimated based on their transcript levels given in publicly available databases (CCLE) [93]. These values were normalized as the ratio of mRNA expression levels in a particular line to those in the HCT116 line. For HCT116, the proportion parameter was 1, indicating that this cell line corresponded to the concentration values found in Table 2, whereas other cell lines exhibited concentrations that varied proportionally according to their expression levels. Due to the fact that we used available expression data of antioxidant genes (which generally, but not always, correlate with protein levels), not actual protein amounts, our results of the H_2_O_2_ level in a steady state after neutralization were presented in arbitrary units. Although this was a limitation of our model, the easy accessibility of transcriptome data allowed us to analyze many cell lines and make predictions based on model results, especially in light of recent genome-scale studies suggesting a stable protein–mRNA ratio reflected by steady-state mRNA levels in human cell lines [98,99]. The original data presented in the study obtained from the mathematical model are openly available in RepOD repository at [https://doi.org/10.18150/ZDFI94].

### Model Equations

Here, we present a set of mathematical model Equations (1)–(7) used in the article. The model contains the following components: (a) Main equation (Equation (1)) of H_2_O_2_ neutralization by neutralizing enzymes through 3 different neutralization pathways with H_2_O_2_ as the state variable, (b) the GSH system, encompassing the GPX and GSR, with GSH/GSSG as state variables (Equations (2) and (3)), and (c) the PRDX/TXN system, which considers the reduced/oxidized species PRDX and TXN as state variables (both couples linked through relationships) (Equations (4)–(7)). In the present model, catalase was also taken into account and appears in equation 1 as an additional ROS scavenger.

(1) $$\frac{{\mathrm{dH}}_{2}O_{2}}{\mathrm{dt}}={\;H}_{2}O_{2\;\mathrm{IN}}\;-\;k_{\mathrm{CAT}}H_{2}O_{2}*\mathrm{CAT}\;-\;k_{\mathrm{GPX}}H_{2}O_{2}*\mathrm{GSH}*\mathrm{GPX}\;-\;k_{\mathrm{PRDX}}H_{2}O_{2}*\mathrm{PRDX}$$

(2) $$\frac{\mathrm{dGSH}}{\mathrm{dt}}=-\;k_{\mathrm{GPX}}H_{2}O_{2}*\mathrm{GSH}*\mathrm{GPX}+k_{\mathrm{GSR}}\mathrm{GSSG}*\mathrm{GSR}$$

(3) $$\frac{\mathrm{dGSSG}}{\mathrm{dt}}={\;k}_{\mathrm{GPX}}H_{2}O_{2}*\mathrm{GSH}*\mathrm{GPX}\;-\;k_{\mathrm{GSR}}\mathrm{GSSG}*\mathrm{GSR}$$

(4) $$\frac{\mathrm{dGSSG}}{\mathrm{dt}}={\;k}_{\mathrm{GPX}}H_{2}O_{2}*\mathrm{GSH}*\mathrm{GPX}\;-{\;k}_{\mathrm{GSR}}\mathrm{GSSG}*\mathrm{GSR}$$

(5) $$\frac{\mathrm{dPRDXox}}{\mathrm{dt}}={\;k}_{\mathrm{PRDX}}H_{2}O_{2}*\mathrm{PRDX}\;-\;k_{\mathrm{TXN}}\mathrm{TXN}*{\mathrm{PRDX}}_{\mathrm{ox}}$$

(6) $$\frac{\mathrm{dTXN}}{\mathrm{dt}}=-\;k_{\mathrm{TXN}}{\mathrm{TXN}*\mathrm{PRDX}}_{\mathrm{ox}}+k_{\mathrm{TXNRD}}{\mathrm{TXN}}_{\mathrm{ox}}*\mathrm{TXNRD}$$

(7) $$\frac{\mathrm{dTXNox}\;}{\mathrm{dt}}=k_{\mathrm{TXN}}\mathrm{TXN}*{\mathrm{PRDX}}_{\mathrm{ox}}{\;-\;k}_{\mathrm{TXNRD}}{\mathrm{TXN}}_{\mathrm{ox}}*\mathrm{TXNRD}$$

In a set of Equations (1)–(7), [PRDX], [PRDX_ox_], [TXN], [TXN_ox_], [GSH], and [GSSG], represent the concentrations of peroxiredoxin, oxidized peroxiredoxin, thioredoxin, oxidized thioredoxin, glutathione, and oxidized glutathione, respectively. We assumed that the total amount of reduced and oxidized forms was constant and typical for the cell. It should be noted that PRDX_TOTAL_ = PRDX + PRDX_ox_, GSH_TOTAL_= GSH + GSSG, and TXN_TOTAL_ = TXN+TXN_ox_. In the implementation, we also assumed that the concentrations of GSR, GPX, TXNRD, and CAT were constant values and dependent on the cell type. Moreover, we assumed that the H_2_O_2_ influx in cells was approximately constant and was influenced by many factors, e.g., a constant influx from mitochondria or peroxisomes. In Equation (1), H_2_O_2IN_ reflects this constant H_2_O_2_ influx; therefore, for the implementation, we used a step function, which was designated as H_2_O_2IN_.

## Figures and Tables

**Table 1:** Radioresistant and radiosensitive lung, breast and colorectal cancer cell lines used in mathematical model of H_2_O_2_ neutralization.

Type of Cancer	Cell Line	Radiosensitive (RS)/ Radioresistant (RR)	Reference
**Lung**	A549	RR	[39]
	H1703	RR	[40]
	H661	RR	[41]
	H1299	RR	[42]
	H1339	RR	[43]
	H292	RR	[44]
	H358	RR	[44]
	H23	RS	[44]
	H441	RS	[45]
	H1650	RS	[46]
	H522	RS	[46]
	HCC827	RS	[40]
	H69	RS	[47]
	H460	RS	[48]
**Breast**	MCF-7	RR	[49,50,51]
	SK-BR-3	RR	[52]
	ZR-751	RR	[53]
	HCC1428	RR	[54]
	T47D	RR	[55,56]
	HS578T	RR	[50]
	UACC-812	RR	[57]
	MDA-MB-175VII	RR	[54]
	MDA-MB-361	RS	[55]
	HCC70	RS	[58]
	MDA-MB-231	RS	[49,51,56]
	BT474	RS	[50,59]
	JIMT-1	RS	[51]
	CAL-51	RS	[60]
	HCC1395	RS	[61]
**Colorectal**	HT115	RR	[62]
	DLD-1	RR	[62]
	Lovo	RR	[62]
	HT29	RR	[62]
	Caco-2	RR	[63,64]
	SW480	RR	[65,66]
	MDST8	RR	[67]
	Colo-201	RS	[62]
	Colo-205	RS	[62]
	Colo-320	RS	[62]
	HCT116	RS	[62]
	SW48	RS	[65,66]

**Table 2:** Initial concentration of enzymes used in the model of H_2_O_2_ neutralization.

Description	Symbol	Value ^1^ [mM]
CAT Concentration	CAT	0.001
PRDX Concentration	PRDX	0.15
TXN Concentration	TXN	0.025
TXNRD Concentration	TXNRD	0.025
GSH Concentration	GSH	3.0
GPX Concentration	GPX	0.05
GSR Concentration	GSR	0.05

^1^ A set of parameters to which the remaining calculated values of expression are referred.

**Table 3:** Parameters used in the model of H_2_O_2_ neutralization.

Description	Symbol	Value [unit]
Rate Constant of CAT	kCAT	0.034 [mM^−1^ ms^−1^]
Rate Constant of PRDX	kPRDX	0.26 [mM^−1^ ms^−1^]
Rate Constant of TXN	kTXN_ox_	0.23 [mM^−1^ ms^−1^]
Rate Constant of TXNRD	kTXNRD	0.31 [mM^−1^ ms^−1^]
Rate Constant of GSR	kGSR	0.08 [mM^−1^ ms^−1^]
Rate Constant of GPX ^1^	kGPX	67 [mM^−2^ ms^−1^]
H_2_O_2_ Influx to the System ^1^	H_2_O_2IN_	10^−5^ [mM ms^−1^]

^1^ Adjusted.

## Data Availability

The original data presented in the study are openly available in RepOD repository at [https://doi.org/10.18150/ZDFI94].
